# Biologic therapies targeting type 2 cytokines are effective at improving asthma symptoms and control—a systematic review and meta-analysis

**DOI:** 10.1016/j.jacig.2024.100374

**Published:** 2024-11-26

**Authors:** Rebecca E. Bignold, Hannah Busby, Jenny Holloway, Aaishah Kasu, Sonia Sian, Jill R. Johnson

**Affiliations:** School of Biosciences, College of Health and Life Sciences, Aston University, Birmingham, United Kingdom

**Keywords:** Asthma, biologics, pharmacotherapy, inflammation, cytokine

## Abstract

**Background:**

Allergic asthma is a highly prevalent chronic inflammatory disease driven by aeroallergen exposure. In severe asthma, the current standard of care does not fully control disease symptoms, indicating an unmet clinical need. Biologic therapies targeting cytokines IL-4, IL-5, and IL-13 have been shown to provide benefits to asthmatic patients over currently existing asthma treatments.

**Objective:**

We sought to review the effects of recently developed biologic therapies for asthma treatment.

**Methods:**

In this meta-analysis, the impact of IL-5 and IL-4/IL-13 biologic inhibitors was critically appraised considering overall lung function, symptom control, and oral corticosteroid use in asthmatic patients. Trials were identified using PubMed, Web of Science, Scopus, and clinicaltrials.gov. Clinical trials assessing severe asthmatic participants older than 12 years were included.

**Results:**

The meta-analysis included 6600 participants from 14 trials published in 2013 to 2020. For IL-5 inhibitors, improvements in FEV_1_ (mean difference [MD], 0.11; 95% CI, 0.11 to 0.12), Asthma Control Questionnaire scores (MD, −0.4; 95% CI, −0.41 to −0.38), annual exacerbation rates (MD, −0.46; 95% CI, −0.48 to −0.45), and oral corticosteroid use (MD, −50; 95% CI, −52.58 to −47.42) favored biologic treatment. Significant improvements in FEV_1_ (MD, 0.11; 95% CI, 0.10 to 0.11), Asthma Control Questionnaire scores (MD, −0.20; 95% CI, −0.22 to −0.18), and annual exacerbation rates (MD, −0.15; 95% CI, −0.16 to −0.14) were also seen with anti–IL-4/IL-13 biologic therapies. However, anti–IL-4/IL-13 inhibitors were associated with more adverse events than placebo (MD, 1.13; 95% CI, 0.97 to 1.3).

**Conclusions:**

Biologic inhibitors targeting T_H_2 cytokines are beneficial for improving overall asthma control.

Asthma is the most prevalent chronic inflammatory disease, affecting nearly 300 million people worldwide.^1^ In the United Kingdom, the treatment of asthma costs between £2912 and £4217 per patient per year,^2^ and despite medical advances, asthma costs the National Health Service at least £1.1 billion every year.^3^ The most common type of asthma is allergic asthma, induced by aeroallergen exposure in susceptible individuals and associated with chronic T_H_2-polarized eosinophilic inflammation.^4^^,^^5^ In a subset of patients with “difficult-to-treat” asthma (estimated to be up to one-third of asthmatic patients), poor symptom control and a high rate of exacerbations are associated with insensitivity to inhaled corticosteroids, the criterion standard medication prescribed for this disease,^6^^,^^7^ indicating an important unmet clinical need in this subgroup of patients as well as an increased economic burden.^8^^,^^9^ Targeted therapies that pinpoint critical disease pathways are a novel approach to tackling severe asthma and improving symptom control.

Allergic asthma occurs following sensitization to aeroallergens, leading to an inflammatory cascade involving airway epithelial cells, type 2 innate lymphoid cells, macrophages, eosinophils, and T_H_2-polarized T_H_ cells.^10^^,^^11^ Over time, this chronic inflammatory response to aeroallergen induces profound changes to the structure of the conducting airways, characterized by increased mucus production, collagen deposition, and airway smooth muscle hyperplasia and hypersensitivity. Collaboratively, allergic inflammation and airway structural changes induce airway hyperreactivity (wheeze), which has an important detrimental effect on quality of life. Lung function changes are measured using spirometry, most often by measuring the FEV_1_, that is, the volume of air expelled from the lungs in 1 second.^12^

IL-5 is one of the most important cytokines involved in allergic asthma. When airway epithelium-derived alarmins or antigen-presenting dendritic cells are activated, type 2 innate lymphoid cells and T_H_2 lymphocytes release IL-5.^13^ IL-5 promotes eosinophil differentiation and maturation from CD34^+^ hematopoietic cells and contributes to eosinophil recruitment by upregulating adhesion molecules including CD11a, CD11b, and CD18.^13^ When activated, IL-5 can moreover upregulate the expression of extracellular matrix components, thereby contributing to airway wall remodeling.^13^ This pathway is strongly associated with severe asthma and its symptoms; it is distinguished by increased eosinophils in the sputum and blood, despite corticosteroid treatment.^14^ Understanding this pathway has enabled researchers to develop anti–IL-5 therapies as novel treatments in IL-5–driven allergic diseases such as severe asthma.

Mepolizumab, benralizumab, and reslizumab are biologics that target the IL-5 pathway to treat severe asthma by impairing IL-5 signaling. Mepolizumab, a humanized N-glycosylated IgG1κ mAb, has a high affinity and specificity for the IL-5Rα chain, thereby blocking IL-5 binding on eosinophils,^15^ suppressing the number of eosinophils in the blood, minimizing exacerbations, and reducing the need for corticosteroids.^16^ Benralizumab is a humanized mAb that contributes to a reduction of eosinophilia via antibody-dependent cell-mediated toxicity. It achieves this by binding to the IL-5Rα chain via the Fab domain on eosinophils, preventing IL-5 from binding to eosinophilic receptors.^17^^,^^18^ In addition to IL-5Rα, benralizumab binds to the fragment crystallizable region of FcγRIIIa location on natural killer cells, subsequently activating them and leading to the release of granzymes and perforins, which induce the apoptosis of the eosinophils, thus reducing the eosinophil population at the site of inflammation.^13^ IL-4 and IL-13 are pleiotropic T_H_2-associated cytokines and are required to induce B-cell class switching to produce IgE.^19^^,^^20^ Moreover, IL-13 has an important role in inducing mucus secretion.^21^^,^^22^ Both cytokines share a common receptor chain, specifically IL-4Rα, which consists of 3 complexes: a type 1 complex and 2 type 2 complexes.^23^, ^24^, ^25^ The type 2 receptor complex is important because both cytokines have the ability to bind to this complex because it is a heterodimeric complex consisting of the IL-4Rα subunit and the IL-13Rα1 subunit. This receptor complex is expressed on many immune cells, such as macrophages, dendritic cells, eosinophils, and B lymphocytes, as well as structural cells, such as endothelial cells, fibroblasts, and airway smooth muscle cells.^26^^,^^27^ The interaction of the cytokines with the IL-4Rα/IL-13Rα1 complex stimulates Janus kinase 1/2 and the tyrosine kinase 2 in the cytoplasm.^26^ The activation of these enzymes results in the phosphorylation of the cytoplasmic domain of the receptor complex to create a site for signal transducer and activator of transcription 6 to bind, where it is phosphorylated and translocated to the nucleus, facilitating the production of type 2 cytokines.^28^ The signaling pathway of this receptor complex is shown in Fig 1. Because of the stimulation of the heterodimeric receptor complex, these cytokines play key roles in the pathogenesis of asthma, because they have the ability to induce many structural and inflammatory changes that are seen in this airway disease. These can include the recruitment of eosinophils, T_H_2-cell activation, and mucous production. IL-4 itself has also been implicated in airway remodeling through the upregulation of extracellular matrix protein synthesis such as collagen and fibronectin.^26^

**Fig 1:**
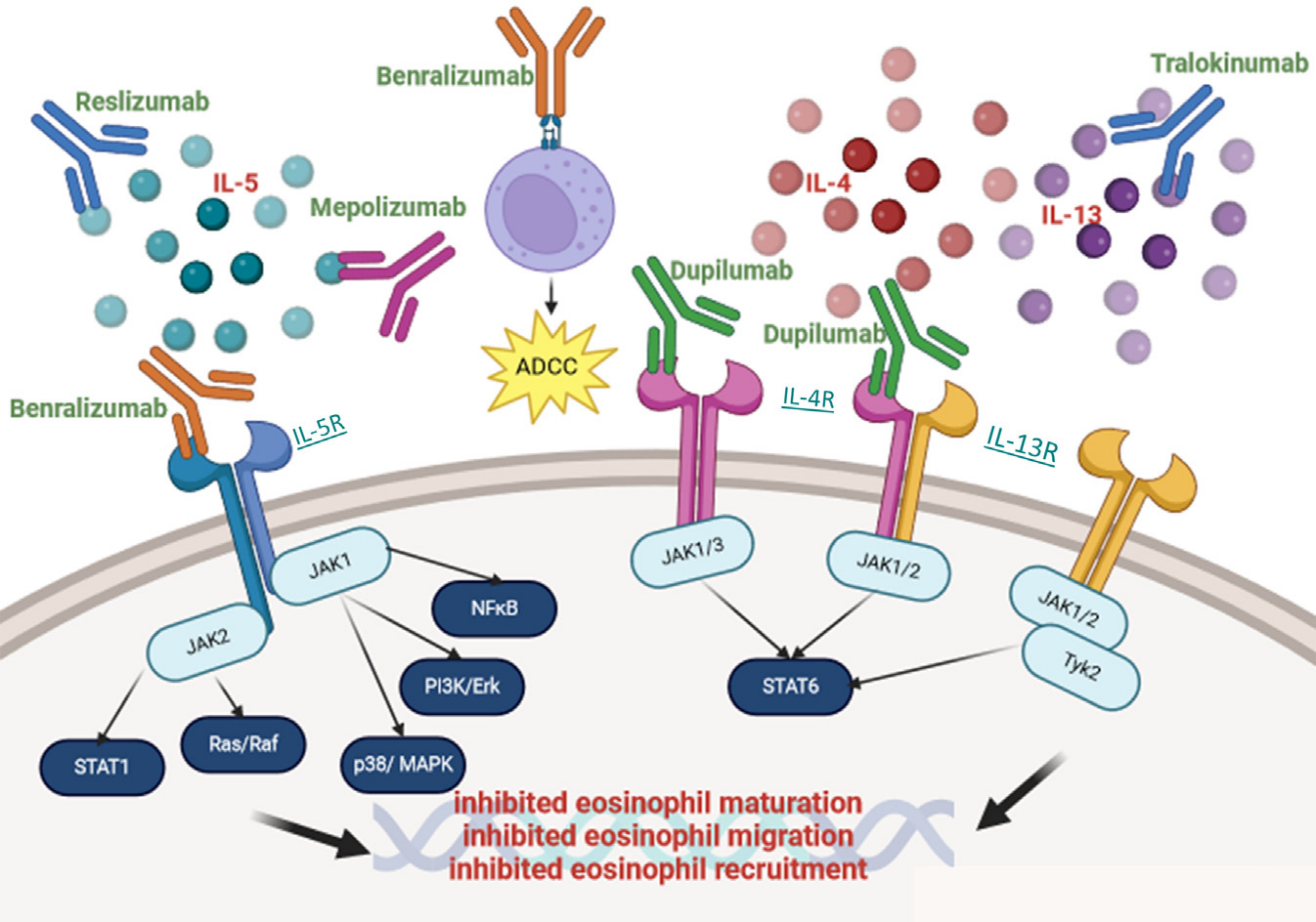
Mechanism of action of biologics targeting IL-5 and IL-4/IL-13. Pathways involved in the inhibition of IL-5, IL-4, and IL-13 signaling by select mAbs. *ADCC*, Antibody-dependent cell-mediated toxicity; *JAK1/2*, Janus kinase 1/2; *MAPK*, mitogen-activated protein kinase; *NF-κB*, nuclear factor κappa B; *STAT1/6*, signal transducer and activator of transcription 1/6. This figure was created using BioRender.

The biologics that target IL-4/IL-13 signaling include dupilumab, pitrakinra, paskolizumab, tralokinumab, lebrikizumab, and anrukinzumab; the latter 3 are anti–IL-13 only, whereas the others are anti–IL-4.^22^ Dupilumab is the most thoroughly studied of these biologics. Dupilumab is an IgG4 mAb targeting IL-4Rα and has the ability to inhibit both IL-4 and IL-13 signaling. It has been investigated for the treatment of several atopic diseases, such as atopic dermatitis, because of its capability to inhibit the T_H_2-mediated responses.^29^^,^^30^ This is thought to occur through the inhibition of IL-4, thus reducing the recruitment and activation of T_H_2 cells, resulting in a reduction of type 2 cytokine production mitigating the inflammation.^31^

Given the increasing incidence of allergic asthma and the poor response to standard therapy in severe disease, new treatment strategies are needed. In this systematic review and meta-analysis, data from clinical studies were critically appraised to identify the impact of anti–IL-5 and anti–IL-4/IL-13 biologic therapy on important disease parameters. The findings show that these novel treatments are beneficial and lead to improvements in a range of clinical outcomes, including lung function, exacerbations, and overall asthma control, highlighting their potential in the treatment of severe asthmatic patients.

## Methods

This systematic review assessed randomized, double-blind clinical trials that included severe eosinophilic asthmatic patients older than 12 years. The trials randomized patients to biologic therapy or a placebo; all participants continued to receive standard treatment.

**Fig 2:**
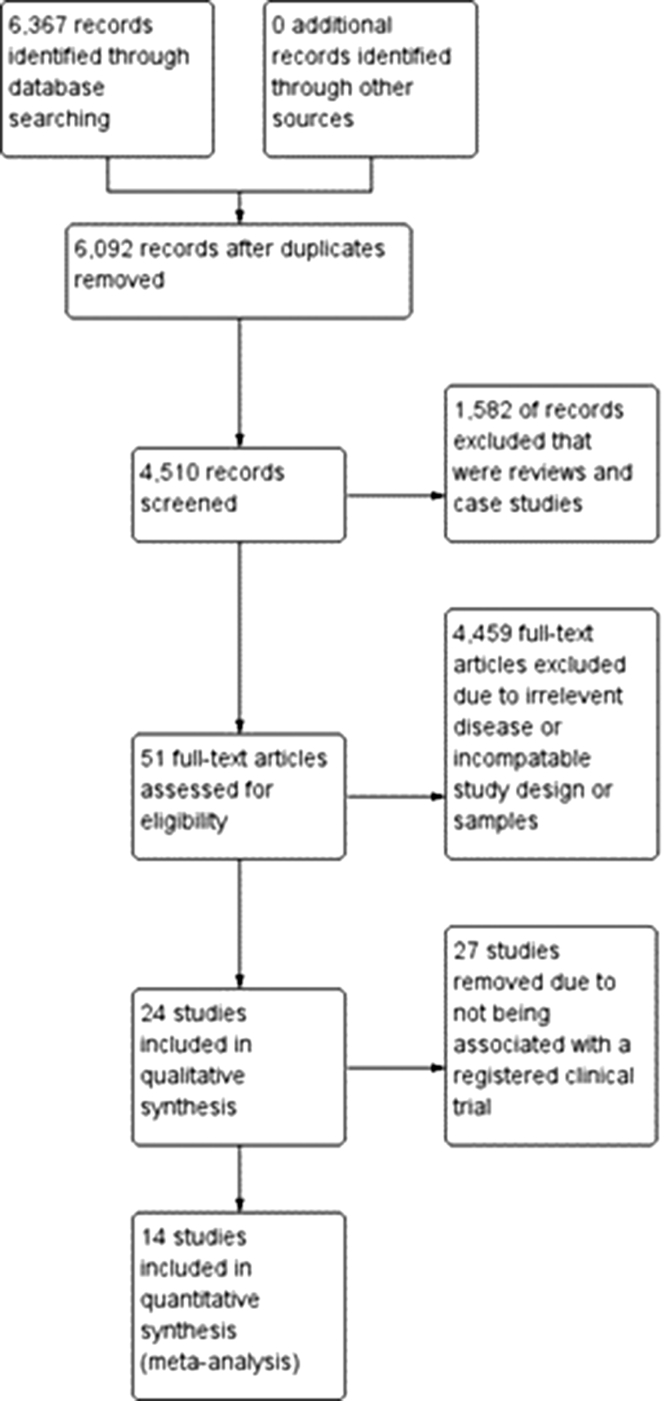
Study flow diagram. This PRISMA flow diagram shows the process of selecting studies for qualitative and quantitative analyses.^32^

The review was carried out following the Preferred Reporting Items for Systematic Reviews and Meta-Analyses (PRISMA) flow diagram shown in Fig 2 and analyzed the use of IL-5 inhibitors and IL-4/IL-13 inhibitors as novel biologic treatments for asthma, compared with current standard therapy.

Most of the clinical trials included in this meta-analysis were phase 3 studies with fairly large cohort sizes. The inclusion criteria consisted of men and women who were older than 12 years. The observations and outcomes included assessments of asthma control (using the Asthma Control Questionnaire [ACQ]) and lung function (measured by FEV_1_). In a subset of studies, asthma exacerbation rates and changes in oral corticosteroid (OCS) use were also assessed. The rate of adverse events associated with these novel biologics was also investigated. Studies performed in animal models of disease as well as review articles were excluded from the qualitative and quantitative analyses.

PubMed, clinicaltrials.gov, Web of Science, and Scopus were used to search for articles and extract the references that emerged. These searches included the terms (IL-4 inhib∗ AND IL-13 Inhib∗ asthma) OR (IL-4 inhib∗ AND IL-13 Inhib∗ asthma AND randomized clinical trials) OR (dupilumab asthma) OR (anti-IL-4 AND anti-IL-13 asthma) OR (IL-4 inhib∗ asthma AND IL-13 inhib∗ asthma), “severe asthma” AND (“IL-4” OR “IL-13” OR “dupilumab” OR “tralokinumab” OR “lebrikizumab” OR “clinical trial”), “anti-IL-5 therapy” AND “IL-5 asthma” AND “anti-IL-5 asthma” AND “IL-5 therapy” and (“IL-5” OR “interleukin 5” OR “inhibitors of IL-5”) AND (“asthma” OR “asthmatic”) AND (“allergy” OR “allergic”) AND (“inflammation” OR “allergic inflammation” OR “inflammatory”). The searches were restricted to studies in English. Following this, the data sets were transferred to Endnote, where duplicate entries were removed. Fig 2 depicts the PRISMA diagram and study selection flow chart.

A meta-analysis was carried out to construct forest plots to summarize the results of all the included studies. When studies assessed multiple drug dosages or different dosage strategies, the highest dose and most represented experimental group was selected for analysis. Similarly, in studies that observed cohorts with differing characteristics (such as different baseline concentrations of blood eosinophils), the group with the highest disease severity was selected. One article was selected that included 2 clinical trials (STRATOS 1 and STRATOS 2, designated as Panettieri 2018a and 2018b, respectively, for distinction).^33^ The basic characteristics of studies included have been collated in Table E1 (in the Online Repository available at www.jaci-global.org).

All included articles were assessed by 3 independent reviewers. Data for the meta-analyses were inputted into the RevMan software (The Cochrane Collaboration, London, United Kingdom) and forest plots were generated. The statistical analysis included calculation of *P* values, 95% CIs, the *I*^2^ statistic to assess heterogeneity, and the *z* statistic to assess the overall effect.

## Results

To assess the suitability of studies selected, a risk of bias table was constructed as each individual study was investigated for several types of bias.

**Fig 3:**
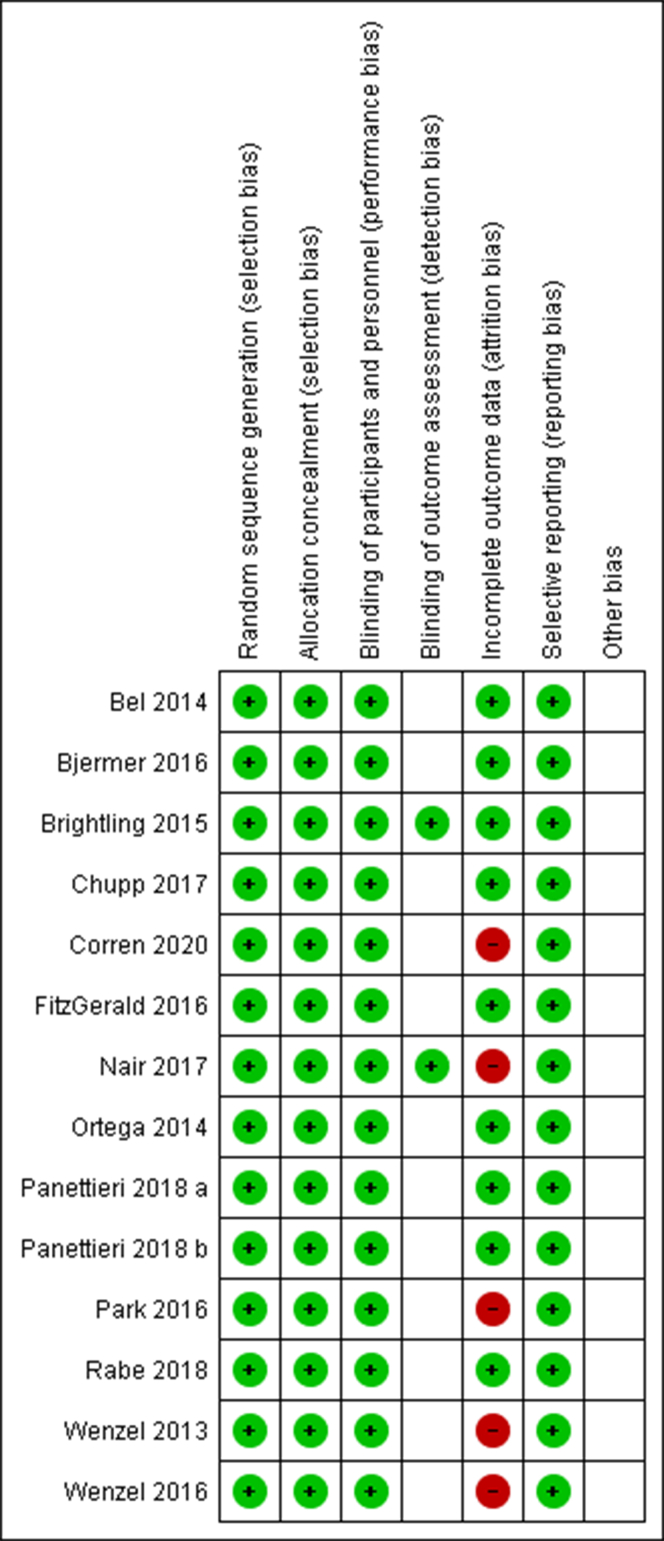
Risk of bias summary. This summary shows the review authors’ judgments about each risk of bias item for each included study: (+) low risk, (−) high risk, and () unclear risk.^32^

Fig 3 shows the potential risk of bias in each study. Selection and performance bias was judged to be appropriate because of all studies being randomized and double-blind. Detection bias was judged to be unclear in most cases because the blinding of assessment was ambiguous. Reporting bias was judged to be of low risk because all studies contained sufficient data on adverse effects. Attrition bias was judged to be of high risk when the reason for uncompleted participation was not mentioned. Overall, these results show a low risk of bias across all studies.

### IL-5 and IL-4/IL-13 inhibitors increase prebronchodilator lung function compared with placebo

One of the key outcomes that was explored in most of the articles selected was the change in FEV_1_ results compared with baseline values taken at the start of the study.

**Fig 4:**
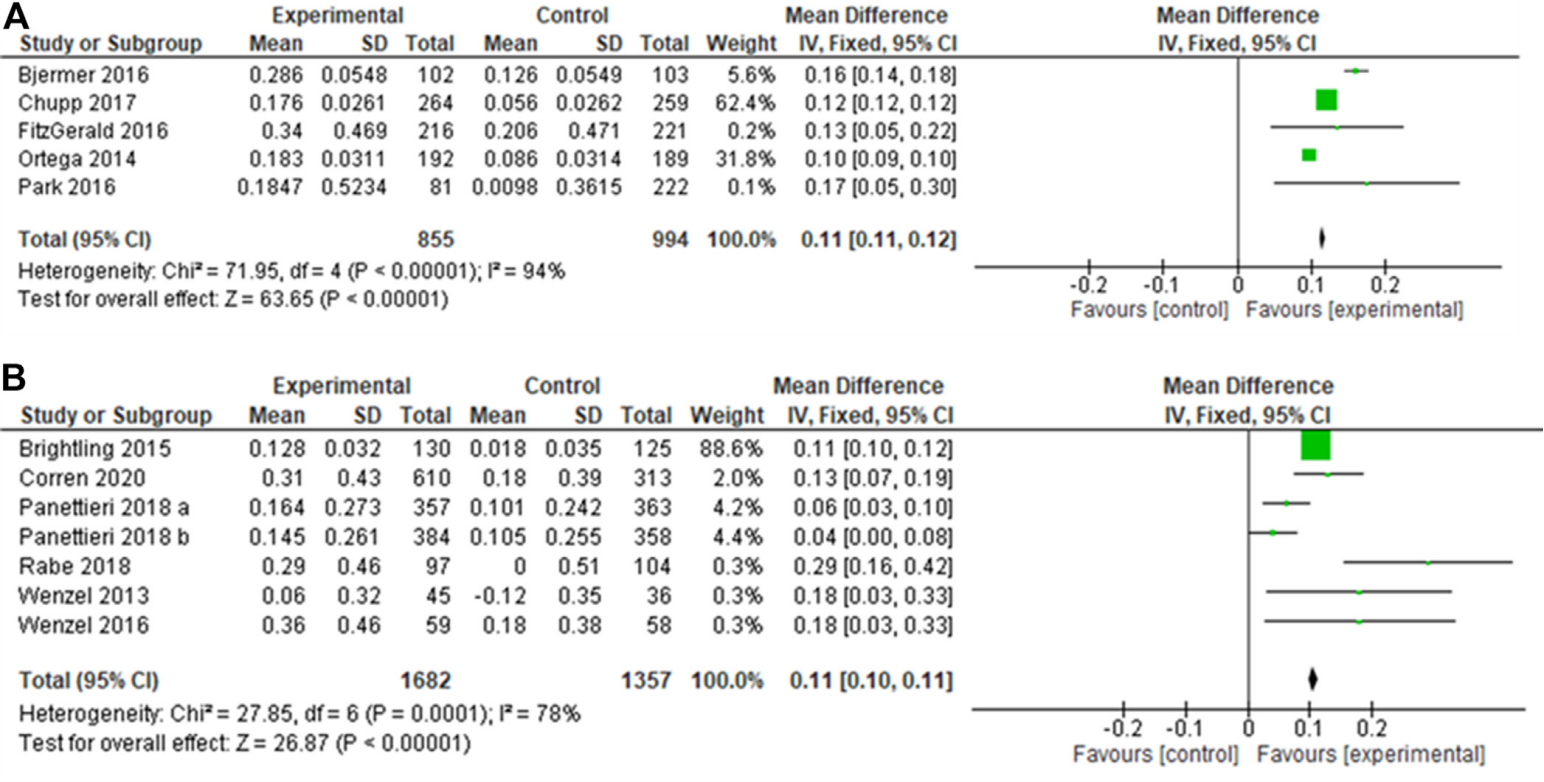
**A** and **B,** Forest plot on lung function improvements. Forest plot of comparison: change in FEV_1_ from baseline using IL-5 inhibitors (Fig 4, *A*) or IL-4/IL-13 inhibitors (Fig 4, *B*) compared with a placebo. FEV_1_ was measured in liters.^32^

Fig 4 shows that both IL-5 and IL-4/IL-13 inhibition significantly increased FEV_1_ and therefore improved lung function compared with the control (*P* < .00001 for both types of treatment). The *I*^2^ values for both types of drugs showed high levels of heterogeneity (94% for IL-5 inhibitors and 78% for IL-4/IL-13 inhibitors). Both types of drugs also showed equal effectiveness in this outcome with a mean difference (MD) of 0.11 for both. The study with the greatest increase in lung function was by Rabe et al,^34^ which highlighted that treatment with dupilumab increased FEV_1_ by 0.29 L compared with an increase of 0 L with the placebo.

### IL-5 and IL-4/IL-13 inhibitors increase perceived asthma control compared with placebo

Asthma control was explored using a standardized questionnaire, that is, the ACQ. Patients were asked to rate several aspects of asthma control on a 7-point scale, with low scores indicating good disease control.

**Fig 5:**
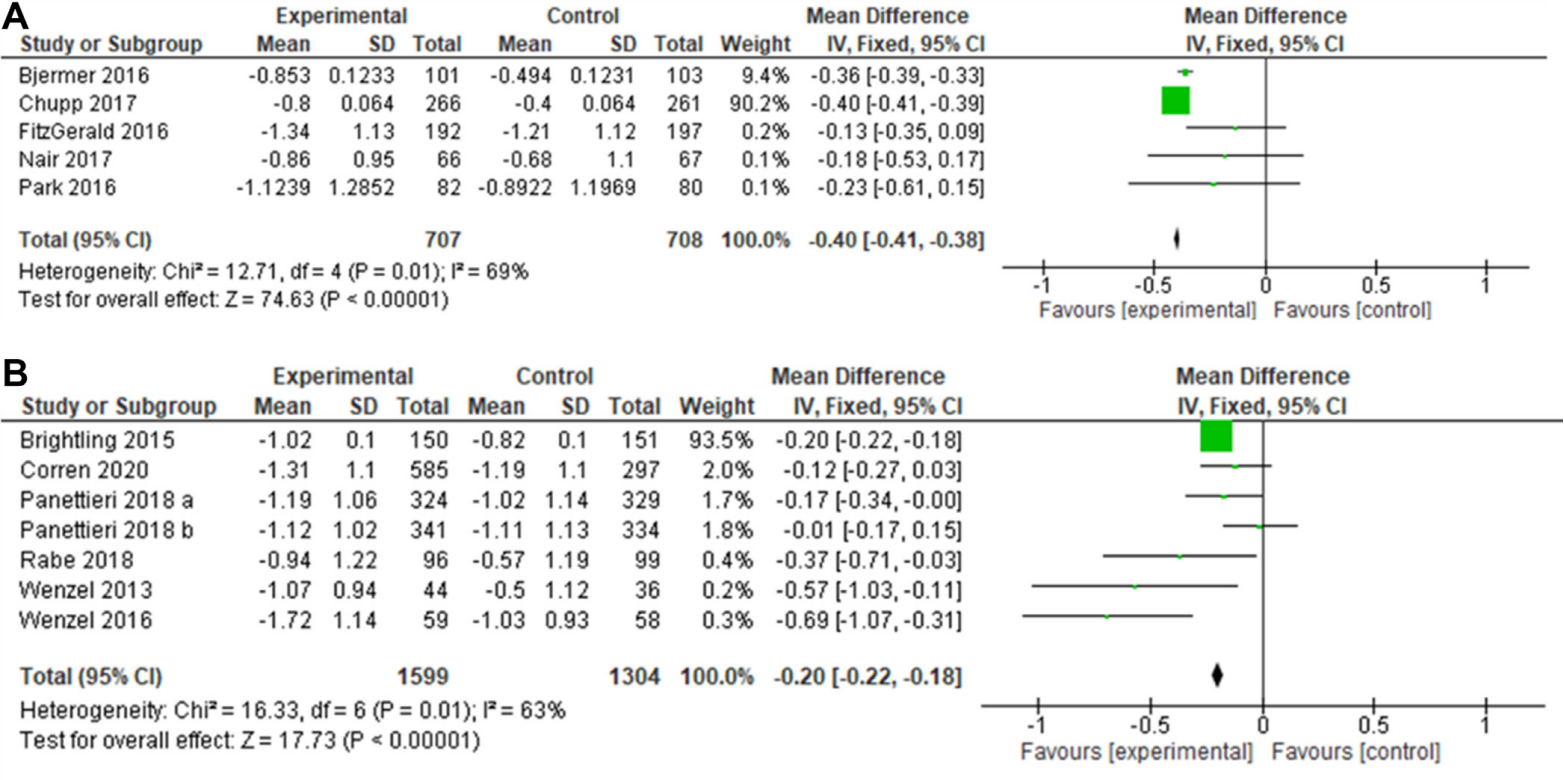
**A** and **B,** Forest plot on ACQ scores. Forest plot of comparison: change in ACQ scores using IL-5 inhibitors (Fig 5, *A*) or IL-4/IL-13 inhibitors (Fig 5, *B*) compared with a placebo. Low scores indicate more controlled asthma.^32^

Fig 5 shows that both IL-5 and IL-4/IL-13 inhibition statistically decreased ACQ scores and therefore improved asthma control compared with the placebo (*P* < .00001 for both types of treatment). The *I*^2^ values for both types of drugs showed moderate levels of heterogeneity (69% for IL-5 inhibitors and 63% for IL-4/IL-13 inhibitors). IL-5 inhibitors seemed to show a more pronounced increase in asthma control than IL-4/IL-13 inhibitors with MDs of −0.4 and −0.2, respectively.

### IL-5 and IL-4/IL-13 inhibitors decrease rate of asthma exacerbations compared with placebo

Asthma exacerbations are defined as a sudden worsening of asthma symptoms that requires medical attention or hospitalization. A way of standardizing the exacerbation rate is to use the annual exacerbation rate, which is calculated from data recorded (or extrapolated for shorter studies) to estimate the number of exacerbations per participant per year.

**Fig 6:**
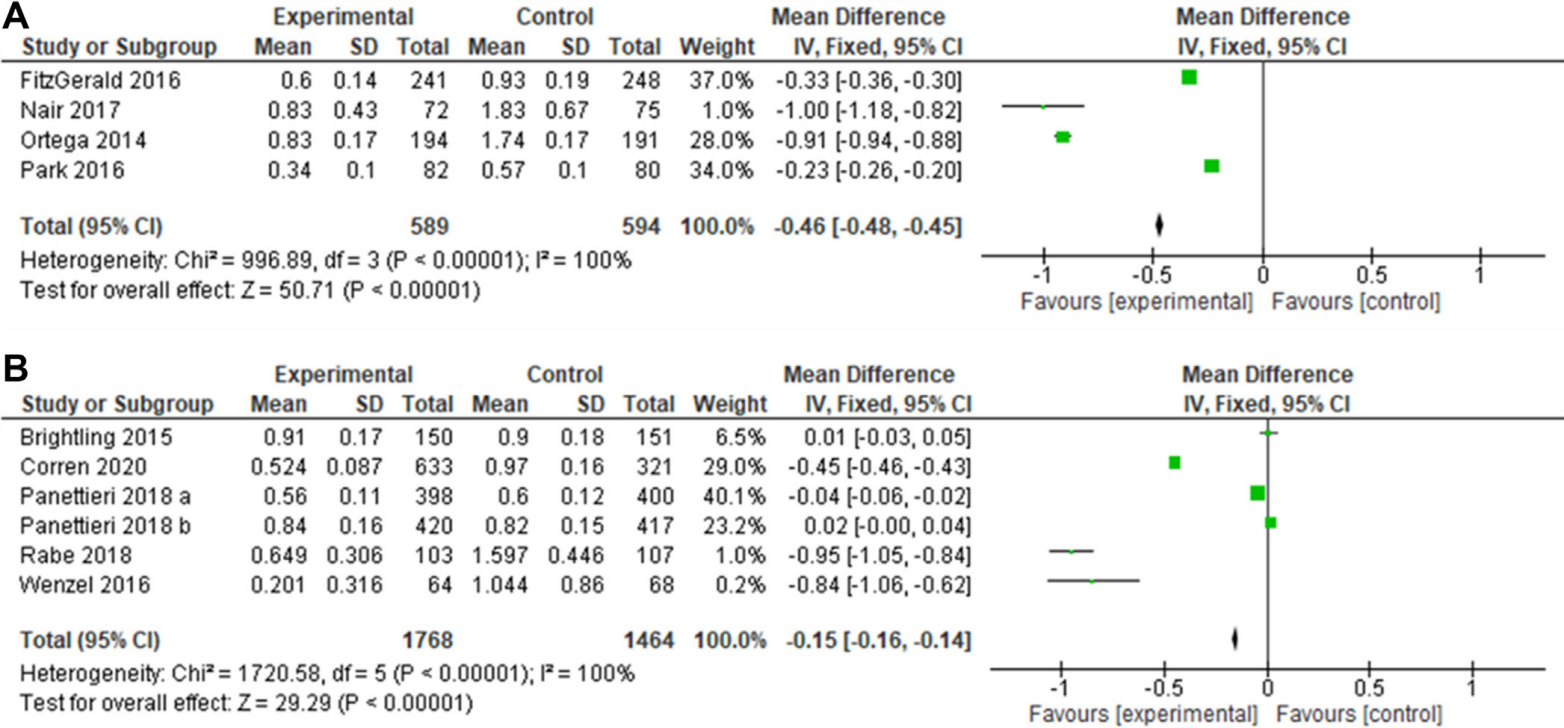
**A** and **B,** Forest plot on annual asthma exacerbation rate. Forest plot of comparison: annual exacerbation rate throughout the experiment using IL-5 inhibitors (Fig 6, *A*) or IL-4/IL-13 inhibitors (Fig 6, *B*) compared with a placebo.^32^

Fig 6 shows that both IL-5 and IL-4/IL-13 inhibition statistically decreased annual exacerbation rates compared with the placebo (*P* < .00001 for both types of treatment). The *I*^2^ values for both types of drugs showed very high levels of heterogeneity (100% for both types of drug), which indicates considerable variation between studies. IL-5 inhibitors were associated with a more pronounced decrease in the exacerbation rate than IL-4/IL-13 inhibitors, with MDs of −0.46 and −0.15, respectively. Some studies, namely, those by Panettieri et al^33^ and Brightling et al,^35^ found higher exacerbation rates in the placebo group than in the experimental group.

### IL-5 inhibitors may allow a reduction in the prescribed OCS dose compared with placebo

One aspect of asthma treatment that was explored by 2 studies was the change in OCS dose. Many of the patients with severe asthma who took part in these studies were prescribed an OCS in addition to traditional inhaled corticosteroids. Because of the adverse effects associated with OCS use, Bel et al^36^ and Nair et al^37^ were interested in whether treatment with an IL-5 inhibitor would allow the prescribed dose of OCS to be lowered.

**Fig 7:**
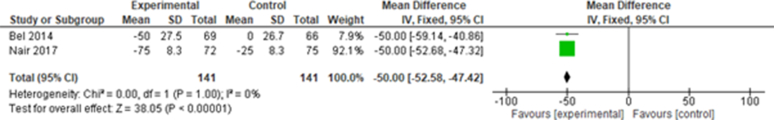
Forest plot on change in OCS dose. Forest plot of comparison: change in OCS dose from baseline using IL-5 inhibitors compared with a placebo.^32^

Fig 7 shows that IL-5 inhibition allowed for a significant reduction in OCS dose compared with the placebo (*P* < .00001). The *I*^2^ values showed no heterogeneity (0%), which indicates a strong similarity between studies. The MD in the 2 studies that included this analysis was −50, indicating a 50% reduction in the prescribed OCS dose. However, because of the small number of studies and participants (only 141 participants were included in this comparison), this outcome should be further investigated.

### IL-5 inhibitors but not IL-4/IL-13 inhibitors may cause less adverse events compared with placebo

Adverse events were reported in all studies included in this analysis, allowing for a broad comparison of the potential negative impact of these novel treatments.

There was a difference in the adverse events observed with each drug type. IL-5 inhibitors were associated with significantly fewer adverse effects than the placebo (MD, 0.069; *P* < .0001), whereas there was no difference in the number of adverse events observed with IL-4/L-13 inhibitors compared with placebo (MD, 1.13; *P* = .38). Heterogeneity was also considerably lower on this outcome for IL-4/IL-13 inhibitors (*I*^2^ = 6%) compared with IL-5 inhibitors (*I*^2^ = 86%). Further details of serious adverse effects reported in the studies are provided in Table E2 (in the Online Repository available at www.jaci-global.org).

## Discussion

Fourteen studies involving more than 6000 participants and 2 different drug types were assessed in this meta-analysis on the impact of biologic therapies targeting T_H_2 cytokines in severe asthmatic participants. The studies had very little risk of bias overall (see Fig 3). All studies were randomized, double-blind studies and included a placebo, presenting a very low risk of selection and performance bias. Reporting bias was also low because of all studies presenting both minor and serious adverse effects, implying their results were honest and truthful. Attrition bias was quite high for some studies in which the reasons for participant withdrawal were not stated. This is significant because it demonstrates that the results of the studies included in the meta-analysis are reliable and able to add to the current understanding of asthma treatment.

The 2 classes of drugs assessed in the meta-analysis focused on 5 drugs: the IL-5 inhibitors mepolizumab, benralizumab, and reslizumab^36^, ^37^, ^38^, ^39^, ^40^, ^41^, ^42^ and the IL-4/IL-13 inhibitors tralokinumab and dupilumab.^33^, ^34^, ^35^^,^^43^, ^44^, ^45^ These were pooled together to increase the sample size of this meta-analysis and to facilitate comparisons between the 2 classes of inhibitors.

One of the most important outcomes reported in these studies was FEV_1_, a common measurement of lung function (shown in Fig 4). In this meta-analysis, all biologic therapies were shown to be effective in improving FEV_1_ before bronchodilation. The increase in FEV_1_ observed in these studies indicated the beneficial effect of these drugs on lung function; this is especially pertinent because some studies have shown that inhaled glucocorticoid therapy does not always have a beneficial impact on FEV_1_.^46^ Both classes of drug investigated here showed an MD of 0.11 on this measure, indicating that both classes were effective at increasing lung function. Studies included in this analysis measured FEV_1_ in liters because this is standard. However, further analysis of measurements to produce *z* scores by comparing measurements to a standard healthy reference value would be more accurate because it eliminates variation due to age or sex.^47^

Fig 5 shows the changes in ACQ scores associated with anti–IL-5 and anti–IL-4/IL-13 therapy. This questionnaire was first developed in 1999 by Juniper et al^48^ to create a global standardized questionnaire that can be used to track changes in asthma control in adults. More recent studies have maintained its effectiveness and have suggested a high correlation with other measures such as fractional exhaled nitrogen oxide or FEV_1_.^49^ This meta-analysis shows that all studies demonstrated a reduction in ACQ scores compared with placebo and therefore an increase in asthma control. The IL-5 inhibitors showed a greater effect than the IL-4/IL-13 inhibitors (MD, −0.4 and −0.2, respectively). Because all treatments were found to significantly increase asthma control (*P* < .00001), it can be concluded that both biologic classes considered here are effective at improving asthma control. Another interesting aspect of this outcome is that all of the placebo groups also experienced an increase in asthma control, although not as pronounced as the experimental groups. This can be seen in Fig 4 as both the experimental and control groups exhibit a “negative” mean, indicating a decrease in score on the ACQ and therefore an increase in asthma control. This highlights the placebo effect because patients reported feeling more controlled despite not receiving additional medication. This may also be due to response bias because the ACQ is self-reported; the self-analysis of patients may lead to a stronger awareness of symptoms and therefore a skewed report.^50^

The impact of anti–IL-5 and anti–IL-4/IL-13 therapy on the annual exacerbation rate is shown in Fig 6. Exacerbations are described as a sudden worsening of symptoms following an environmental exposure and are linked to mortality. Thus, reducing the rate of exacerbations is central to managing asthma.^51^, ^52^, ^53^, ^54^ The annual exacerbation rate is a standardized measure used in measuring exacerbations in studies of variable duration and has been shown to be associated with lung function.^55^ All studies involving IL-5 inhibitors showed a decrease in the annual exacerbation rate (with an average MD of −0.46); however, this varied between studies (*I*^2^ = 100%), with effects ranging from −0.1 to −0.23. This differs from the IL-4/IL-13 inhibitor results as, although there was an overall improvement in the exacerbation rate, 2 studies showed a marginal increase in the exacerbation rate (study by Panettieri et al,^33^ MD, 0.02; study by Brightling et al,^35^ MD, 0.01); both of these studies assessed tralokinumab. However, another separate study by Panettieri that also assessed tralokinumab had a relatively high MD (−0.04).^33^ Further studies assessing the impact of tralokinumab on the annual exacerbation rate are warranted.

A secondary outcome that was investigated in this analysis was the reduction in OCS dose. This was explored by only 2 studies, but was included because of its clinical importance. Severe asthmatic patients are often prescribed OCSs as an add-on therapy, although their long-term use is associated with many undesirable side effects, including psychological and musculoskeletal complications.^56^ Therefore, new drugs that aim to reduce the reliance on OCSs would be beneficial. Both of the studies included in this analysis that explored this outcome showed that there was a significant decrease in the OCS dose prescribed at the end of the study when patients were also given an IL-5 inhibitor (MD, −50; *P* < .00001; *I*^2^ = 0%). This was calculated in the studies as a percentage reduction in the OCS maintenance dose during the 4 weeks of the study compared with the participants’ initial prescribed baseline dose. Both of these studies showed this result and therefore there was no heterogeneity within this sample. However, the sample size is small compared with the other outcomes described in this analysis, with 141 patients in each of the experimental and placebo groups. Interestingly, these studies investigated different IL-5 inhibitors, that is, mepolizumab and benralizumab, which suggests that this effect may be a common effect of this drug class; however, additional studies should be performed before firm conclusions are drawn. The impact of IL-4/IL-13 inhibitors should also be explored in relation to this outcome.

**Fig 8:**
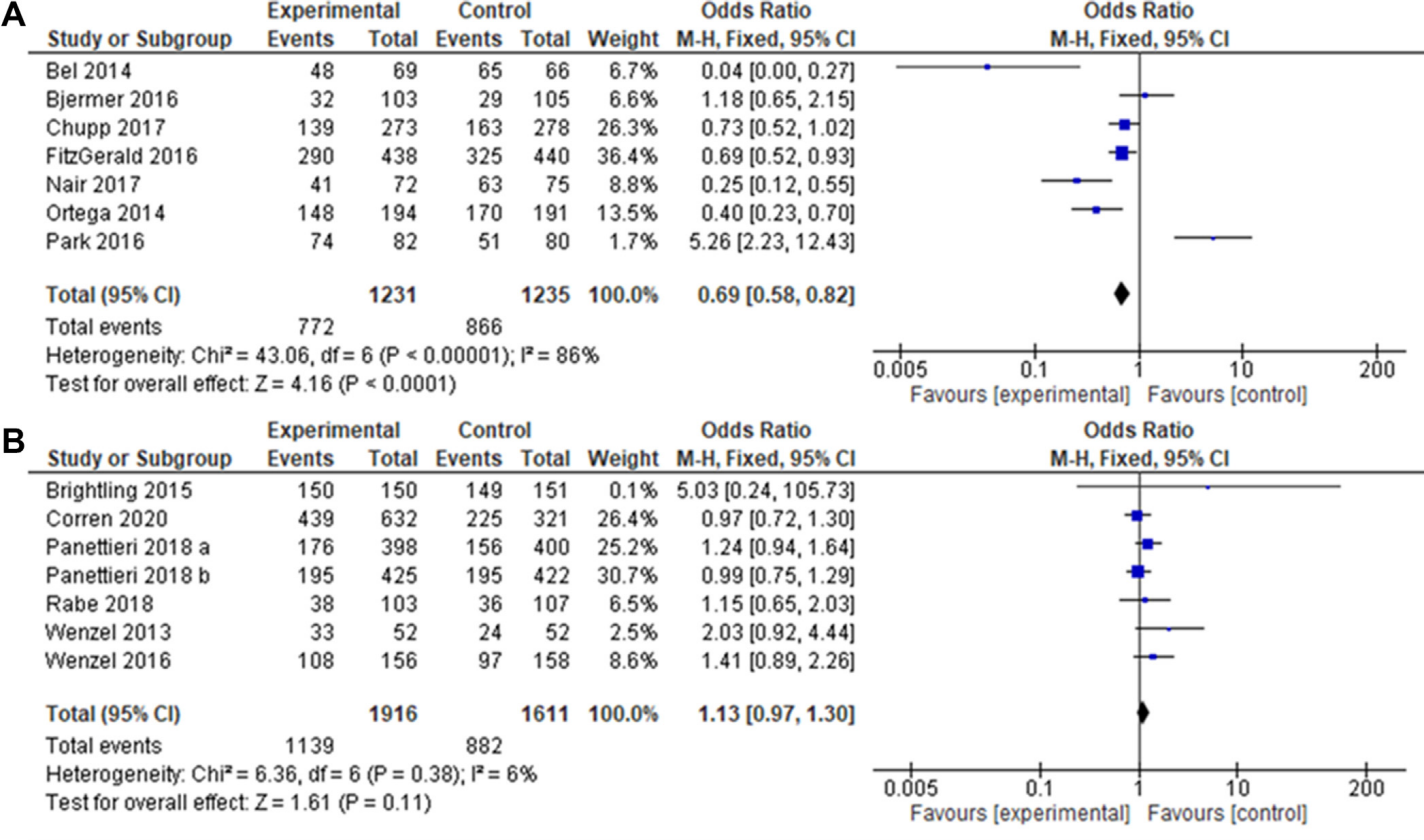
**A** and **B,** Forest plot on adverse effects. Forest plot of comparison: adverse events recorded throughout the studies using IL-5 inhibitors (Fig 8, *A*) or IL-4/IL-13 inhibitors (Fig 8, *B*) compared with a placebo.^32^

Because all clinical trials are required to report adverse events, this outcome was explored in this analysis (Fig 8). Severe and nonsevere adverse events were pooled to maintain consistency. An interesting difference in this outcome was between IL-5 inhibitors and IL-4/IL-13 inhibitors, as patients given IL-5 inhibitors generally experienced fewer adverse events than the placebo group; there was a similar number of adverse events seen with IL-4/IL-13 inhibitors compared with placebo (MD, 0.69 and 1.13, with *P* < .0001 and *P* = .11, respectively). Two studies involving an IL-5 inhibitor^38^^,^^42^ and 4 studies involving an IL-4/IL-13 inhibitor^33^^,^^35^^,^^44^^,^^45^ showed more adverse events with biologic therapy than with the placebo. These studies tested 2 different IL-5 inhibitors and 2 different IL-4/IL-13 inhibitors and so it cannot be concluded that these increased adverse effects are unique to certain drugs (Table E2). Because neither of the drug types showed a statistically higher frequency of adverse events than the placebo group, it can be concluded that these drugs are unlikely to induce adverse events and are therefore safe for use.

The 14 studies included had methodologies that differed significantly; an approximation of the efficacy of biologic therapy can therefore be derived by assessing the overall results. Furthermore, because these studies varied in administration schedules and drug doses, some of the results were inconsistent. Sources of heterogeneity included variability in the participants, interventions, and outcomes (ie, clinical heterogeneity) as well as variability in study design and methods of assessment (ie, methodological heterogeneity). The disparities in these studies should be acknowledged before coming to firm conclusions, and future studies should consider using consistent methods. In addition, a greater range of outcomes could be explored, including additional physiological tests such as fractional exhaled nitrogen oxide and blood eosinophil concentrations, as well as measures assessing airway remodeling. It may be beneficial to further investigate the effect of biologics by completing a network meta-analysis. This was not completed on the data included in this study because of the inclusion of studies that did not investigate 3 or more treatments or conditions.^57^

All the anti–IL-5 and anti–IL-4/IL-13 therapies tested in the included studies showed beneficial effects in terms of lung function, asthma control, and reducing the rate of exacerbations. Further testing into the efficacy of targeting these inflammatory pathways should be pursued to develop more targeted and efficacious therapies for severe asthma.

**Clinical implications**

This meta-analysis demonstrates that current biologics targeting either IL-5 or IL-4/IL-13 are effective in treating allergic asthma because of the increase in ACQ scores and FEV_1_ following treatment.

## Disclosure statement

Disclosure of potential conflict of interest: The authors declare that they have no relevant conflicts of interest.
