# Supplementary material for: Biologic therapies targeting type 2 cytokines are effective at improving asthma symptoms and control—a systematic review and meta-analysis
Source: J Allergy Clin Immunol Glob. 2024 Nov 26;4(1):100374. doi: 10.1016/j.jacig.2024.100374 (PMC11751513; doi:10.1016/j.jacig.2024.100374)
Supplement: Supplementary Tables [file mmc1.docx]

Supplementary Table S1 - Collated basic characteristics of all studies

|  |  |  |  |  |  | **Sex** |  | **Ethnicity** |  |  |  |  |
| --- | --- | --- | --- | --- | --- | --- | --- | --- | --- | --- | --- | --- |
| **Study** | **Intervention** | **Outcomes** | **Group** | **Number** | **Age (mean)** | **Male** | **Female** | **White** | **Black** | **Asian** | **Mixed Race** | **Other** |
| Bel 2014 | Mepolizumab | OCS dose, adverse events | Placebo | 66 | 49.9 | 36 | 30 | 61 | 0 | 2 | 1 | 2 |
|  |  |  | Experimental | 69 | 49.8 | 25 | 44 | 67 | 0 | 1 | 1 | 0 |
| Bjermer 2016 | Reslizumab | FEV_1_, ACQ, adverse events | Placebo | 105 | 44.2 | 43 | 62 | 85 | 7 | 0 | 0 | 13 |
|  |  |  | Experimental | 106 | 43.0 | 44 | 62 | 90 | 5 | 2 | 0 | 9 |
| Brightling 2015 | Tralokinumab | FEV_1_, ACQ, exacerbation rate, adverse events | Placebo | 76 | 48.8 | 25 | 51 | Unknown |  |  |  |  |
|  |  |  | Experimental | 150 | 49.7 | 50 | 100 | Unknown |  |  |  |  |
| Chupp 2017 | Mepolizumab | FEV_1_, ACQ, adverse events | Placebo | 277 | 52.1 | 101 | 176 | 259 | 7 | 0 | 0 | 11 |
|  |  |  | Experimental | 274 | 49.8 | 125 | 149 | 252 | 8 | 4 | 0 | 10 |
| Corren 2020 | Dupilumab | FEV_1_, ACQ, exacerbation rate, adverse events | Placebo | 321 | 48.2 | 103 | 218 | 273 | 12 | 33 | 0 | 3 |
|  |  |  | Experimental | 633 | 47.7 | 239 | 394 | 529 | 21 | 79 | 0 | 4 |
| FitzGerald 2016 | Benralizumab | FEV_1_, ACQ, exacerbation rate, adverse events | Placebo | 440 | 48.8 | 176 | 264 | Unknown |  |  |  |  |
|  |  |  | Experimental | 425 | 50.0 | 155 | 270 | Unknown |  |  |  |  |
| Nair 2017 | Benralizumab | ACQ, exacerbation rate, OCS dose, adverse events | Placebo | 75 | 49.9 | 27 | 48 | Unknown |  |  |  |  |
|  |  |  | Experimental | 72 | 50.2 | 32 | 40 | Unknown |  |  |  |  |
| Ortega 2014 | Mepolizumab | FEV_1_, exacerbation rate, adverse events | Placebo | 191 | 49.2 | 84 | 107 | 148 | 3 | 38 | 2 | 0 |
|  |  |  | Experimental | 191 | 50.0 | 85 | 106 | 151 | 6 | 33 | 1 | 0 |
| Panettieri 2018 a | Tralokinumab | FEV_1_, ACQ, exacerbation rate, adverse events | Placebo | 400 | 51.4 | 135 | 265 | 288 | 14 | 55 | 0 | 43 |
|  |  |  | Experimental | 398 | 49.4 | 146 | 252 | 285 | 21 | 53 | 0 | 39 |
| Panettieri 2018 b | Tralokinumab | FEV_1_, ACQ, exacerbation rate, adverse events | Placebo | 417 | 48.0 | 127 | 290 | 281 | 24 | 88 | 0 | 24 |
|  |  |  | Experimental | 420 | 47.3 | 144 | 276 | 283 | 27 | 83 | 0 | 27 |
| Park 2016 | Benralizumab | FEV_1_, ACQ, exacerbation rate, adverse events | Placebo | 440 | 48.8 | 176 | 264 | Unknown |  |  |  |  |
|  |  |  | Experimental | 425 | 50.0 | 155 | 270 | Unknown |  |  |  |  |
| Rabe 2018 | Dupilumab | FEV_1_, ACQ, exacerbation rate, adverse events | Placebo | 107 | 50.7 | 42 | 65 | 100 | 1 | 2 | 0 | 4 |
|  |  |  | Experimental | 103 | 51.9 | 41 | 62 | 97 | 4 | 0 | 0 | 2 |
| Wenzel 2013 | Dupilumab | FEV_1_, ACQ, adverse events | Placebo | 52 | 41.6 | 26 | 26 | 38 | 9 | 3 | 0 | 2 |
|  |  |  | Experimental | 52 | 37.8 | 26 | 26 | 45 | 5 | 1 | 0 | 1 |
| Wenzel 2016 | Dupilumab | FEV_1_, ACQ, exacerbation rate, adverse events | Placebo | 158 | 49 | 54 | 104 | 119 | 9 | 25 | 0 | 5 |
|  |  |  | Experimental | 157 | 47.5 | 54 | 103 | 129 | 5 | 22 | 0 | 1 |

Supplementary Table S2 - Reported instances of “serious” adverse effects from all studies

| **Study** | **Intervention** | **Adverse Effect** | **Placebo** | **Experimental** |
| --- | --- | --- | --- | --- |
| Bel 2014 | Mepolizumab | Pneumonia | 0 | 3 |
|  |  | Chronic sinusitis | 0 | 1 |
|  |  | Urinary tract infection | 1 | 0 |
|  |  | Post gastric syndrome | 1 | 0 |
|  |  | Hypokalaemia | 0 | 1 |
|  |  | Fistula | 0 | 1 |
|  |  | Basosquamous carcinoma | 1 | 0 |
|  |  | Prostate cancer | 1 | 0 |
|  |  | Prostatitis | 1 | 0 |
|  |  | Asthma | 7 | 0 |
| Bjermer 2016 | Reslizumab | Acute myocardial infarction | 1 | 0 |
|  |  | Pneumonia | 0 | 1 |
|  |  | Sinusitis | 0 | 1 |
|  |  | Rib fracture | 0 | 1 |
|  |  | Road traffic accident | 0 | 1 |
|  |  | Asthma | 0 | 3 |
| Brightling 2015 | Tralokinumab | Myocarditis | 0 | 1 |
|  |  | Abdominal pain | 1 | 0 |
|  |  | Diabetic gastropathy | 1 | 0 |
|  |  | Diverticulum | 1 | 0 |
|  |  | Femoral hernia | 1 | 0 |
|  |  | Hiatus hernia | 1 | 0 |
|  |  | Large intestine perforation | 1 | 0 |
|  |  | Abdominal abscess | 1 | 0 |
|  |  | Abdominal infection | 1 | 0 |
|  |  | Amoebiasis | 1 | 0 |
|  |  | Bronchitis | 0 | 2 |
|  |  | Diverticulitis | 1 | 0 |
|  |  | Gastroenteritis | 1 | 1 |
|  |  | Pharyngotonsillitis | 1 | 0 |
|  |  | Pneumonia | 2 | 3 |
|  |  | Pneumonia pneumococcal | 0 | 1 |
|  |  | Ankle fracture | 0 | 1 |
|  |  | Ligament sprain | 1 | 0 |
|  |  | Testicular injury | 1 | 0 |
|  |  | Dehydration | 1 | 1 |
|  |  | Diabetes mellitus | 1 | 0 |
|  |  | Arthralgia | 0 | 1 |
|  |  | Intervertebral disc protrusion | 0 | 1 |
|  |  | Myalgia | 0 | 1 |
|  |  | Osteoarthritis | 2 | 0 |
|  |  | Basal cell carcinoma | 1 | 0 |
|  |  | Squamous cell carcinoma of the cervix | 0 | 1 |
|  |  | Acute renal failure | 1 | 0 |
|  |  | Dysfunctional uterine bleeding | 1 | 0 |
|  |  | Menometrorrhagia | 1 | 0 |
|  |  | Allergic sinusitis | 1 | 0 |
|  |  | Asthma | 6 | 9 |
|  |  | Pulmonary embolism | 0 | 1 |
|  |  | Angioedema | 1 | 0 |
|  |  | Peripheral artery thrombosis | 0 | 1 |
| Chupp 2017 | Mepolizumab | Anaemia | 1 | 0 |
|  |  | Myocardial ischemia | 1 | 0 |
|  |  | Palpitations | 0 | 1 |
|  |  | Adrenal insufficiency | 1 | 0 |
|  |  | Hiatus hernia | 0 | 1 |
|  |  | Non-cardiac chest pain | 1 | 0 |
|  |  | Pyrexia | 1 | 0 |
|  |  | Allergic granulomatous angiitis | 0 | 1 |
|  |  | Hypersensitivity | 1 | 0 |
|  |  | Appendicitis | 0 | 1 |
|  |  | Bronchitis bacterial | 1 | 0 |
|  |  | Catheter site infection | 0 | 1 |
|  |  | Gastroenteritis rotavirus | 1 | 0 |
|  |  | Hemophilus infection | 1 | 0 |
|  |  | Laryngitis | 0 | 1 |
|  |  | Localized infection | 1 | 0 |
|  |  | Lung infection | 1 | 0 |
|  |  | Sinusitis | 1 | 0 |
|  |  | Staphylococcal bacteraemia | 0 | 1 |
|  |  | Staphylococcal infection | 0 | 1 |
|  |  | Post-procedural complication | 2 | 0 |
|  |  | Clavicle fracture | 0 | 1 |
|  |  | Fibula fracture | 1 | 0 |
|  |  | Meniscus injury | 0 | 1 |
|  |  | Tibia fracture | 1 | 0 |
|  |  | Blood glucose increased | 0 | 1 |
|  |  | Dehydration | 1 | 0 |
|  |  | Rheumatic disorder | 1 | 0 |
|  |  | Dizziness | 0 | 1 |
|  |  | Urethral stenosis | 0 | 1 |
|  |  | Asthma | 9 | 3 |
|  |  | Acute respiratory failure | 1 | 0 |
|  |  | Nasal polyps | 0 | 1 |
|  |  | Angioedema | 1 | 0 |
|  |  | Atopic dermatitis | 1 | 0 |
|  |  | Hypertension | 1 | 0 |
|  |  | Hypotension | 1 | 0 |
|  |  | Subclavian vein thrombosis | 0 | 1 |
|  |  | Vasculitis | 1 | 0 |
| Corren 2020 | Dupilumab | Eosinophilia | 0 | 1 |
|  |  | Neutropenia | 1 | 0 |
|  |  | Acute myocardial infarction | 0 | 2 |
|  |  | Angina pectoris | 0 | 1 |
|  |  | Atrial fibrillation | 0 | 1 |
|  |  | Atrioventricular block (2^nd^ degree) | 0 | 1 |
|  |  | Congestive cardiac failure | 0 | 1 |
|  |  | Cardio-respiratory arrest | 0 | 2 |
|  |  | Ischaemic cardiomyopathy | 0 | 1 |
|  |  | Myocardial ischaemia | 0 | 1 |
|  |  | Retinal detachment | 0 | 1 |
|  |  | Retinal tear | 0 | 1 |
|  |  | Abdominal hernia | 0 | 1 |
|  |  | Diverticulum | 1 | 0 |
|  |  | Haemorrhoids | 1 | 0 |
|  |  | Inguinal hernia | 1 | 0 |
|  |  | Large intestine polyp | 1 | 0 |
|  |  | Oesophageal motility disorder | 0 | 1 |
|  |  | Oesophageal ulcer | 0 | 1 |
|  |  | Pancreatitis acute | 0 | 1 |
|  |  | Strangulated umbilical hernia | 0 | 1 |
|  |  | Umbilical hernia | 0 | 1 |
|  |  | Chest pain | 0 | 1 |
|  |  | Impaired healing | 0 | 1 |
|  |  | Injection site erythema | 0 | 1 |
|  |  | Injection site inflammation | 0 | 1 |
|  |  | Injection site oedema | 0 | 1 |
|  |  | Non-cardiac chest pain | 1 | 0 |
|  |  | Cholecystitis | 0 | 1 |
|  |  | Cholecystitis acute | 0 | 2 |
|  |  | Cholecystitis chronic | 0 | 1 |
|  |  | Anaphylactic | 0 | 1 |
|  |  | Abscess | 0 | 1 |
|  |  | Bronchitis | 0 | 2 |
|  |  | Chronic sinusitis | 0 | 1 |
|  |  | Clostridium difficile colitis | 0 | 1 |
|  |  | Diverticulitis | 1 | 0 |
|  |  | Gastroenteritis | 1 | 1 |
|  |  | Hepatitis A | 0 | 1 |
|  |  | Medical site infection | 0 | 1 |
|  |  | Pneumonia | 2 | 4 |
|  |  | Pyelonephritis | 0 | 1 |
|  |  | Tick-borne viral encephalitis | 0 | 1 |
|  |  | Upper respiratory tract infection | 1 | 0 |
|  |  | Concussion | 1 | 0 |
|  |  | Contusion | 0 | 1 |
|  |  | Facial bones fracture | 0 | 1 |
|  |  | Fall | 0 | 2 |
|  |  | Femur fracture | 0 | 1 |
|  |  | Fibula fracture | 1 | 0 |
|  |  | Lower limb fracture | 0 | 1 |
|  |  | Pneumothorax traumatic | 1 | 0 |
|  |  | Postoperative thoracic procedure complication | 1 | 0 |
|  |  | Rib fracture | 1 | 0 |
|  |  | Road traffic accident | 2 | 0 |
|  |  | Spinal compression fracture | 0 | 1 |
|  |  | Ulna fracture | 0 | 1 |
|  |  | Upper limb fracture | 0 | 1 |
|  |  | Wrist fracture | 0 | 1 |
|  |  | Intervertebral disc protrusion | 1 | 0 |
|  |  | Musculoskeletal chest pain | 0 | 1 |
|  |  | Osteoarthritis | 2 | 2 |
|  |  | Pathological fracture | 0 | 1 |
|  |  | Adenocarcinoma of colon | 0 | 1 |
|  |  | Basal cell carcinoma | 0 | 1 |
|  |  | Breast cancer | 1 | 0 |
|  |  | Malignant melanoma | 0 | 2 |
|  |  | Uterine leiomyoma | 1 | 1 |
|  |  | Loss of consciousness | 0 | 1 |
|  |  | Migraine | 1 | 0 |
|  |  | Syncope | 1 | 1 |
|  |  | Abortion threatened | 1 | 0 |
|  |  | Ectopic pregnancy | 1 | 0 |
|  |  | pregnancy | 0 | 1 |
|  |  | Anxiety | 1 | 1 |
|  |  | Depression | 0 | 1 |
|  |  | Urinary incontinence | 0 | 1 |
|  |  | Cervical cyst | 0 | 1 |
|  |  | Asthma | 5 | 7 |
|  |  | Eosinophilic pneumonia chronic | 0 | 1 |
|  |  | Haemoptysis | 1 | 0 |
|  |  | Nasal polyps | 0 | 1 |
|  |  | Pleurisy | 0 | 1 |
|  |  | Pneumothorax spontaneous | 0 | 1 |
|  |  | Pulmonary embolism | 0 | 1 |
|  |  | Respiratory depression | 0 | 1 |
|  |  | Hypotension | 1 | 0 |
|  |  | Thrombophlebitis superficial | 0 | 1 |
| FitzGerald 2016 | Benralizumab | Anaemia | 1 | 0 |
|  |  | Acute myocardial infarction | 1 | 1 |
|  |  | Aortic valve stenosis | 1 | 0 |
|  |  | Atrioventricular block complete | 1 | 0 |
|  |  | Cardiac arrest | 1 | 0 |
|  |  | Cardiac failure congestive | 0 | 1 |
|  |  | Coronary artery disease | 1 | 0 |
|  |  | Myocardial infarction | 1 | 1 |
|  |  | Myocardial ischaemia | 2 | 0 |
|  |  | Supraventricular tachycardia | 1 | 0 |
|  |  | Gastritis | 1 | 0 |
|  |  | Inguinal hernia | 0 | 1 |
|  |  | Large intestine polyp | 0 | 1 |
|  |  | Rectal polyp | 0 | 1 |
|  |  | Chest pain | 0 | 1 |
|  |  | Non-cardiac chest pain | 1 | 0 |
|  |  | Cholecystitis acute | 1 | 0 |
|  |  | Cholelithiasis | 0 | 1 |
|  |  | Cholecystitis chronic | 0 | 1 |
|  |  | Hepatitis alcoholic | 0 | 1 |
|  |  | Drug hypersensitivity | 1 | 0 |
|  |  | Bacterial infection | 0 | 1 |
|  |  | bronchitis | 0 | 1 |
|  |  | Chronic sinusitis | 0 | 1 |
|  |  | Liver abscess | 1 | 0 |
|  |  | Pneumonia | 4 | 2 |
|  |  | Pneumonia bacterial | 3 | 2 |
|  |  | Pseudomonas bronchitis | 1 | 0 |
|  |  | Pseudomonas infection | 1 | 0 |
|  |  | Respiratory tract infection bacterial | 0 | 1 |
|  |  | Sepsis | 0 | 1 |
|  |  | Sinusitis | 0 | 1 |
|  |  | Humerus fracture | 1 | 0 |
|  |  | Injury | 1 | 0 |
|  |  | Joint injury | 1 | 0 |
|  |  | Road traffic accident | 0 | 1 |
|  |  | Thermal burn | 1 | 0 |
|  |  | Epiphysiolysis | 1 | 0 |
|  |  | Intervertebral disc disorder | 1 | 1 |
|  |  | Jaw cyst | 0 | 1 |
|  |  | Muscular weakness | 0 | 1 |
|  |  | Osteoarthritis | 1 | 1 |
|  |  | Rheumatoid arthritis | 0 | 1 |
|  |  | Rotator cuff syndrome | 1 | 0 |
|  |  | Spinal osteoarthritis | 0 | 1 |
|  |  | Spondylolisthesis | 1 | 0 |
|  |  | Breast cancer | 1 | 0 |
|  |  | Gallbladder cancer | 0 | 1 |
|  |  | Gastric cancer | 0 | 1 |
|  |  | Thyroid adenoma | 1 | 0 |
|  |  | Uterine leiomyoma | 0 | 1 |
|  |  | Cerebrovascular accident | 1 | 0 |
|  |  | Lumbar radiculopathy | 1 | 0 |
|  |  | Sciatica | 1 | 0 |
|  |  | Syncope | 0 | 1 |
|  |  | Transient ischaemic attack | 1 | 0 |
|  |  | Suicide | 0 | 1 |
|  |  | Calculus ureteric | 0 | 1 |
|  |  | Nephrolithiasis | 0 | 2 |
|  |  | Benign prostatic hyperplasia | 1 | 0 |
|  |  | Asthma | 23 | 21 |
|  |  | Hyperventilation | 1 | 0 |
|  |  | Nasal polyps | 1 | 0 |
|  |  | Nasal turbinate hypertrophy | 0 | 1 |
|  |  | Pleural effusion | 0 | 1 |
|  |  | Parakeratosis | 1 | 0 |
|  |  | Urticaria | 0 | 1 |
|  |  | Aortic stenosis | 1 | 0 |
|  |  | Hypertension | 1 | 0 |
|  |  | Hypertensive crisis | 0 | 1 |
|  |  | Phlebitis | 1 | 0 |
|  |  | Venous thrombosis | 1 | 0 |
| Nair 2017 | Benralizumab | Atrial flutter | 1 | 0 |
|  |  | Pericarditis | 1 | 0 |
|  |  | Umbilical hernia | 0 | 1 |
|  |  | Adverse drug reaction | 0 | 1 |
|  |  | Hypersensitivity | 0 | 1 |
|  |  | Atypical pneumonia | 1 | 0 |
|  |  | Chronic sinusitis | 1 | 0 |
|  |  | Colonic abscess | 1 | 0 |
|  |  | Influenza | 2 | 0 |
|  |  | Pneumonia | 3 | 0 |
|  |  | Pneumonia staphylococcal | 1 | 0 |
|  |  | Urinary tract infection bacterial | 0 | 1 |
|  |  | Urosepsis | 0 | 1 |
|  |  | Intervertebral disc protrusion | 0 | 1 |
|  |  | Nephrolithiasis | 0 | 1 |
|  |  | Asthma | 4 | 3 |
|  |  | Sleep apnoea syndrome | 1 | 0 |
|  |  | Status asthmaticus | 3 | 0 |
|  |  | Dermatitis atopic | 0 | 1 |
| Ortega 2014 | Mepolizumab | Intracranial lipoma | 1 | 0 |
|  |  | Gastrointestinal motility disorder | 0 | 1 |
|  |  | Oesophageal spasm | 1 | 0 |
|  |  | Hypersensitivity | 1 | 0 |
|  |  | Bronchitis | 2 | 0 |
|  |  | Clostridium difficile infection | 1 | 0 |
|  |  | Postoperative wound infection | 1 | 0 |
|  |  | Viral upper respiratory tract infection | 0 | 1 |
|  |  | Vulval abscess | 1 | 0 |
|  |  | Contusion | 1 | 0 |
|  |  | Fractured coccyx | 0 | 1 |
|  |  | Heat stroke | 1 | 0 |
|  |  | Rib fracture | 0 | 1 |
|  |  | Road traffic accident | 1 | 0 |
|  |  | Tendon rupture | 0 | 1 |
|  |  | Intervertebral disc protrusion | 1 | 0 |
|  |  | Spinal osteoarthritis | 1 | 0 |
|  |  | Epilepsy | 1 | 0 |
|  |  | Sciatica | 0 | 1 |
|  |  | Major depression | 1 | 0 |
|  |  | Nephrolithiasis | 1 | 0 |
|  |  | Calculus ureteric | 1 | 0 |
|  |  | Calculus urethral | 1 | 0 |
|  |  | Renal colic | 1 | 0 |
|  |  | Asthma | 14 | 9 |
|  |  | Pulmonary fibrosis | 1 | 0 |
|  |  | Angioedema | 1 | 0 |
| Panettieri 2018 a |  | Anaemia | 1 | 1 |
|  |  | Lymphadenopathy | 1 | 0 |
|  |  | Acute myocardial infarction | 2 | 1 |
|  |  | Angina pectoris | 1 | 0 |
|  |  | Angina unstable | 0 | 1 |
|  |  | Arrythmia | 1 | 0 |
|  |  | Atrial fibrillation | 0 | 1 |
|  |  | Cardiac failure | 0 | 1 |
|  |  | Cardiac failure acute | 0 | 1 |
|  |  | Cardiac failure congestive | 0 | 1 |
|  |  | Coronary artery disease | 0 | 1 |
|  |  | Myocardial infarction | 1 | 0 |
|  |  | Pericarditis | 0 | 1 |
|  |  | Sinus node dysfunction | 1 | 0 |
|  |  | Cataract | 1 | 0 |
|  |  | Retinal detachment | 0 | 1 |
|  |  | Diarrhoea | 1 | 0 |
|  |  | Erosive duodenitis | 0 | 1 |
|  |  | Gastroesophageal reflux disease | 1 | 0 |
|  |  | Swollen tongue | 0 | 1 |
|  |  | Umbilical hernia | 1 | 0 |
|  |  | Cholecystitis acute | 0 | 1 |
|  |  | Cholelithiasis | 0 | 2 |
|  |  | Appendicitis | 0 | 1 |
|  |  | Bronchitis | 1 | 0 |
|  |  | Diarrhoea infectious | 0 | 1 |
|  |  | Erysipelas | 0 | 1 |
|  |  | Gastroenteritis | 1 | 0 |
|  |  | Herpes zoster | 1 | 0 |
|  |  | Pneumonia | 4 | 3 |
|  |  | Pyelonephritis | 0 | 1 |
|  |  | Pyelonephritis acute | 1 | 0 |
|  |  | Facial bones fracture | 1 | 0 |
|  |  | Fall | 0 | 1 |
|  |  | Humerus fracture | 0 | 2 |
|  |  | Joint dislocation | 0 | 1 |
|  |  | Femur fracture | 0 | 1 |
|  |  | Laceration | 1 | 0 |
|  |  | Meniscus injury | 0 | 1 |
|  |  | Post-traumatic pain | 1 | 0 |
|  |  | Increased blood pressure | 0 | 1 |
|  |  | Dyslipidaemia | 0 | 1 |
|  |  | Hypokalaemia | 0 | 1 |
|  |  | Hypovolaemia | 1 | 0 |
|  |  | Arthralgia | 1 | 0 |
|  |  | Rheumatoid arthritis | 1 | 0 |
|  |  | Spinal pain | 0 | 1 |
|  |  | Trigger finger | 1 | 0 |
|  |  | Basal cell carcinoma | 0 | 1 |
|  |  | Cervix carcinoma | 0 | 1 |
|  |  | Uterine cancer | 1 | 0 |
|  |  | Radiculopathy | 1 | 0 |
|  |  | Transient ischaemic attack | 0 | 2 |
|  |  | Carpal tunnel syndrome | 1 | 0 |
|  |  | Dizziness | 0 | 1 |
|  |  | Haemorrhagic stroke | 1 | 0 |
|  |  | Ischaemic stroke | 0 | 1 |
|  |  | Vertebrobasilar insufficiency | 0 | 1 |
|  |  | Depression | 0 | 1 |
|  |  | Metrorrhagia | 0 | 1 |
|  |  | Prostatitis | 1 | 0 |
|  |  | Asthma | 25 | 12 |
|  |  | Atelectasis | 0 | 1 |
|  |  | Dyspnoea | 1 | 0 |
|  |  | Pharyngeal oedema | 0 | 1 |
|  |  | Pulmonary embolism | 0 | 1 |
|  |  | Angioedema | 0 | 1 |
|  |  | Hypertension | 1 | 0 |
| Panettieri 2018 b |  | Angina pectoris | 1 | 0 |
|  |  | Angina unstable | 1 | 0 |
|  |  | Atrial fibrillation | 0 | 1 |
|  |  | Cardiac failure | 1 | 0 |
|  |  | Myocardial infarction | 0 | 1 |
|  |  | Colitis ulcerative | 1 | 0 |
|  |  | Constipation | 1 | 0 |
|  |  | Dyspepsia | 0 | 1 |
|  |  | Haematochezia | 1 | 0 |
|  |  | Oesophageal ulcer | 1 | 0 |
|  |  | Oesophagitis | 1 | 0 |
|  |  | Umbilical hernia | 1 | 0 |
|  |  | Oedema peripheral | 0 | 1 |
|  |  | Cholecystitis acute | 0 | 1 |
|  |  | Eosinophilic granulomatosis with polyangiitis | 1 | 0 |
|  |  | Appendicitis | 1 | 0 |
|  |  | Hepatitis A | 1 | 0 |
|  |  | Laryngitis viral | 1 | 0 |
|  |  | Osteomyelitis | 0 | 1 |
|  |  | Otitis media acute | 0 | 1 |
|  |  | Pneumonia | 3 | 4 |
|  |  | Pulmonary tuberculosis | 0 | 1 |
|  |  | Tracheobronchitis | 0 | 1 |
|  |  | Urinary tract infection | 1 | 0 |
|  |  | Urosepsis | 0 | 1 |
|  |  | Humerus fracture | 0 | 1 |
|  |  | Lumbar vertebral fracture | 0 | 1 |
|  |  | Tendon rupture | 0 | 1 |
|  |  | Alanine aminotransferase increased | 0 | 1 |
|  |  | Aspartate aminotransferase increased | 0 | 1 |
|  |  | Obesity | 1 | 0 |
|  |  | Type 2 diabetes mellitus | 0 | 1 |
|  |  | Patellofemoral pain syndrome | 0 | 1 |
|  |  | Rectal cancer | 1 | 0 |
|  |  | Vascular headache | 1 | 0 |
|  |  | Suicidal ideation | 0 | 1 |
|  |  | Urinary incontinence | 1 | 0 |
|  |  | Adnexa uteri pain | 1 | 0 |
|  |  | Ovarian cyst | 0 | 1 |
|  |  | Uterine haemorrhage | 1 | 0 |
|  |  | Asthma | 23 | 17 |
|  |  | Bronchitis chronic | 0 | 1 |
|  |  | Nasal polyps | 1 | 0 |
|  |  | Vitiligo | 0 | 1 |
| Park 2016 | Benralizumab | Anaemia | 1 | 0 |
|  |  | Acute myocardial infarction | 1 | 1 |
|  |  | Aortic valve stenosis | 1 | 0 |
|  |  | Atrioventricular block complete | 1 | 0 |
|  |  | Cardiac arrest | 1 | 0 |
|  |  | Cardiac failure congestive | 0 | 1 |
|  |  | Coronary artery disease | 1 | 0 |
|  |  | Myocardial infarction | 1 | 1 |
|  |  | Myocardial ischaemia | 2 | 0 |
|  |  | Supraventricular tachycardia | 1 | 0 |
|  |  | Gastritis | 1 | 0 |
|  |  | Inguinal hernia | 0 | 1 |
|  |  | Large intestine polyp | 0 | 1 |
|  |  | Rectal polyp | 1 | 0 |
|  |  | Chest pain | 0 | 1 |
|  |  | Non-cardiac chest pain | 1 | 0 |
|  |  | Cholecystitis acute | 1 | 0 |
|  |  | Cholecystitis chronic | 0 | 1 |
|  |  | Cholelithiasis | 0 | 1 |
|  |  | Hepatitis alcoholic | 0 | 1 |
|  |  | Drug hypersensitivity | 1 | 0 |
|  |  | Bacterial infection | 0 | 1 |
|  |  | Bronchitis | 0 | 1 |
|  |  | Chronic sinusitis | 0 | 1 |
|  |  | Cytomegalovirus hepatitis | 1 | 0 |
|  |  | Liver abscess | 1 | 0 |
|  |  | Pneumonia | 4 | 2 |
|  |  | Pneumonia bacterial | 3 | 2 |
|  |  | Pseudomonas bronchitis | 1 | 0 |
|  |  | Pseudomonas infection | 1 | 0 |
|  |  | Respiratory tract infection bacterial | 0 | 1 |
|  |  | Sepsis | 0 | 1 |
|  |  | Sinusitis | 1 | 0 |
|  |  | Humerus fracture | 1 | 0 |
|  |  | Injury | 1 | 0 |
|  |  | Joint injury | 1 | 0 |
|  |  | Road traffic accident | 0 | 1 |
|  |  | Thermal burn | 1 | 0 |
|  |  | Epiphysiolysis | 1 | 0 |
|  |  | Intervertebral disc disorder | 1 | 1 |
|  |  | Jaw cyst | 0 | 1 |
|  |  | Muscular weakness | 0 | 1 |
|  |  | Osteoarthritis | 1 | 1 |
|  |  | Rheumatoid arthritis | 0 | 1 |
|  |  | Rotator cuff syndrome | 1 | 0 |
|  |  | Spinal osteoarthritis | 0 | 1 |
|  |  | Spondylolisthesis | 1 | 0 |
|  |  | Breast cancer | 1 | 0 |
|  |  | Gallbladder cancer | 0 | 1 |
|  |  | Gastric cancer | 0 | 1 |
|  |  | Thyroid adenoma | 1 | 0 |
|  |  | Uterine leiomyoma | 0 | 1 |
|  |  | Cerebrovascular accident | 1 | 0 |
|  |  | Lumbar radiculopathy | 1 | 0 |
|  |  | Sciatica | 1 | 0 |
|  |  | Syncope | 0 | 1 |
|  |  | Transient ischaemic attack | 1 | 0 |
|  |  | Suicide | 0 | 1 |
|  |  | Calculus ureteric | 0 | 1 |
|  |  | Nephrolithiasis | 0 | 2 |
|  |  | Benign prostatic hyperplasia | 1 | 0 |
|  |  | Asthma | 23 | 21 |
|  |  | Hyperventilation | 1 | 0 |
|  |  | Nasal polyps | 1 | 0 |
|  |  | Nasal turbinate hypertrophy | 0 | 1 |
|  |  | Pleural effusion | 0 | 1 |
|  |  | Parakeratosis | 1 | 0 |
|  |  | Urticaria | 0 | 1 |
|  |  | Aortic stenosis | 1 | 0 |
|  |  | Hypertension | 1 | 0 |
|  |  | Hypertensive crisis | 0 | 1 |
|  |  | Phlebitis | 1 | 0 |
|  |  | Venous thrombosis | 1 | 0 |
| Rabe 2018 | Dupilumab | Eosinophilia | 0 | 2 |
|  |  | Pneumonia | 0 | 1 |
|  |  | Respiratory tract infection | 0 | 1 |
|  |  | Acetabulum fracture | 0 | 1 |
|  |  | Foreign body aspiration | 0 | 1 |
|  |  | Type 2 diabetes mellitus | 1 | 0 |
|  |  | Gastrointestinal stromal tumour | 1 | 0 |
|  |  | Asthma | 3 | 3 |
|  |  | Asthmatic crisis | 1 | 0 |
|  |  | Chylothorax | 0 | 1 |
|  |  | Pneumonia aspiration | 0 | 1 |
|  |  | Pneumothorax | 0 | 1 |
|  |  | Pulmonary mass | 0 | 1 |
| Wenzel 2013 | Dupilumab | Pneumonia | 1 | 0 |
|  |  | Ankle fracture | 1 | 0 |
|  |  | Gun shot wound | 1 | 0 |
|  |  | Bipolar disorder | 0 | 1 |
|  |  | Asthma | 1 | 0 |
|  |  | Pneumothorax | 1 | 0 |
| Wenzel 2016 | Dupilumab | Eosinophilia | 0 | 1 |
|  |  | Large intestine polyp | 1 | 0 |
|  |  | Subileus | 0 | 1 |
|  |  | Cholecystitis | 0 | 1 |
|  |  | Anaphylactic reaction | 0 | 1 |
|  |  | Appendicitis | 1 | 1 |
|  |  | Gastroenteritis | 0 | 2 |
|  |  | Herpes zoster | 1 | 0 |
|  |  | Pneumonia | 0 | 1 |
|  |  | Pneumonia bacterial | 0 | 1 |
|  |  | Comminuted fracture | 1 | 0 |
|  |  | Procedural intestinal perforation | 0 | 1 |
|  |  | Blood pressure increased | 0 | 1 |
|  |  | Colon cancer | 1 | 0 |
|  |  | Pregnancy | 0 | 1 |
|  |  | Uterine prolapse | 0 | 1 |
|  |  | Asthma | 4 | 1 |
|  |  | Eczema | 0 | 1 |
